# Supplementary material for: Cell death and biomass reduction in biofilms of multidrug resistant extended spectrum β-lactamase-producing uropathogenic Escherichia coli isolates by 1,8-cineole
Source: PLoS One. 2020 Nov 5;15(11):e0241978. doi: 10.1371/journal.pone.0241978 (PMC7644070; doi:10.1371/journal.pone.0241978)
Supplement: Attachment — Submitted filename: Galvan R1 - Response to Reviewers.docx [file pone.0241978.s001.docx]

Estela M. Galvan, Ph D
Lab. of Bacterial Pathogenesis

Centro de Estudios Biomedicos, Biotecnologicos,

Ambientales y Diagnostico (CEBBAD)

Universidad Maimonides
Hidalgo 775

C1405BWE-Buenos Aires, Argentina

May 06, 2020

Dear Monica Cartelle Gestal, PhD

Academic Editor

PLOS ONE

Please find enclosed our revised version of the manuscript number PONE-D-20-04063 entitled “Cell death and biomass reduction in biofilms of multidrug resistant extended spectrum β-lactamase-producing uropathogenic *Escherichia coli* isolates by 1,8-cineole” for your consideration. It has been revised and changes have been made for each specific comment of the reviewers, as addressed below (modifications are indicated, referring to line numbers in the Marked Up Manuscript file).

**Reviewer #1**

***General comment:*** Vazquez and colleagues present a manuscript that highlights an important issue - antibiotic resistance. With growing resistance to currently available therapeutics - natural compounds are of growing interest as potential biofilm inhibitors.

The manuscript is well written - however I have some concerns about the methodology used and the associated explanation for the chosen methods.

***Author response to general comment:*** We now better explain the rational of the methodology used (see L248-251; L316-318; L324-327; L340-349; L353-354).

***Specific comment #1:*** Why did the authors choose a 3 day biofilm assay? Response to antimicrobial strategies in bacterial populations can be observed at the transcript level (RNA) after ~30 minutes treatment, by growing biofilms for 3 days - my concern at this small scale is that cells within the biofilm are under stress from depleting nutrients and oxygen, and therefore will impact any live/dead assessment being made for the inhibitor.

A time course with treatment would be a good way to test how the inhibitor's efficacy is impacted by depleting biofilm health.

***Author response #1:*** We now better explain in the text the reason for choosing a 3 day-old biofilm (to assess the phytochemical antibiofilm activity over a mature robust biofilm with a substantial biomass, as showed by crystal violet assay) (see L248-250 and L278).

We agree with the reviewer´s comment regarding that cells within 3-d-old biofilms could be under stress for depleting nutrients and oxygen. We now discuss the possibility that the inhibitor´s efficacy could be impacted by this situation and, therefore, that future work is needed to analyze this possibility (see L400-403, L406-408). In addition, results reported by other group, that applied the phytochemical carvacrol at different stages of biofilm development, has been included in the Discussion (see L403-406). These authors found that the compound was more biocidal during early biofilm development compared to mature biofilms.

***Specific comment #2:*** Were flow experiments only performed on dispersed cells? These are likely to be live as they are active - flow should be performed on biofilm cells also to calculate quantity of live/dead. This was not clear in the manuscript.

***Author response #2:*** We now better explain the rational for the experiments to analyse cell viability after 1,8-c treatment on cells detached from the biofilms (by cfu counting and live/dead staining followed by flow cytometry) (see L339-363). On the other hand, we now re-write the paragraph corresponding to assessment of bacterial viability of surface-attached cells by confocal microscopy, to clearly explain that quantification of live/dead cells into biofilms was performed by confocal microscopy followed by COMSTSAT quantitative analysis. (see L316-327).

***Specific comment #3***- 1,8 - cineole may be acting as a dispersal agent - what are the potential health consequences of this in a patient? This should be discussed by the authors. Also additional dispersal assays would strengthen the manuscript. Is motility involved?

***Author response #3:*** We now better explain the effect of 1,8-cineole as partially disrupting the biofilm and causing detachment of mainly dead cells (see L340-364). Nevertheless, because we detected a minor amount of detached cells still alive, we now discuss the importance to further investigate the possible synergistic interactions of 1,8-cineole with common antibiotics to make the phytochemical treatment more efficient in a patient (see L424-430). Besides, we believe that further exploration of the biofilm detachment events caused by the phytochemical is beyond the scope of the present work.

**Reviewer #2**

***General comments:*** This paper's major issue is that the English is not of sufficient quality to review. I believe that the experiments conducted and the methods used are likely sufficient to address the question but at this time the paper must be largely re-written. Due to this I have been unable to fully review the paper but this was my review of the parts I could review.

***Author response to general comment:*** We revised the whole manuscript for proper English usage, several parts of the text has been re-written and we consider that its quality has now been improved.

***Specific comment #1:*** I dislike the term essential oil in a scientific paper. It does not convey a clear description of what the product is. Clearer terms such as plant oil. It can also be misappropriated by alternative medicine proponents. The authors are I assume not working under the guise that the aroma of the oil is in anyway contributing to the antibacterial nature of the oil. If that hold true then the use of ‘essential oil’ should be avoided.

***Author response #1:*** As suggested, the term “essential oil” has been changed by “plant volatile oil” or “volatile oil” all along the manuscript ( see L28,83,87,90,431,453).

***Specific comment #2:*** In terms of scientific questions, the authors layout the following approach. Using the plant extracted oil a substantial reduction in viable cells was observed (a 3-fold log reduction). This was result was obtained in both antibiotic resistant and sensitive cells. This is then expanded to say that there is a reduction in biomass. The remaining biomass was then subjected to confocal analysis of live/dead staining and showed part of the remaining adhered biomass was actually dead. Cell no longer adhered we found to be mostly dead by flow cytometry analysis.

The use of statistics seems reudementary but without access to the raw data (as required by PLOS one) it is hard to determine. I would argue that given your hypothesis and previous work a two tailed test is not correct, you are working under the hypothesis that the plant extract will reduce the biofilm and or cell viability.

***Author response #2:*** As the reviewer requested, we now provide access to all the raw data through the Figshare repository (https://doi.org/10.6084/m9.figshare.c.4964399.v1). According with the reviewer´s comment, we revised the statistics used and now a one-tailed Student´s t-test was applied to our data (see L202 and test results showed in the repository).

We thank the reviewers and editors for their constructive comments and suggestions. We believe that the revised manuscript is now acceptable for publication in PlosOne.

Sincerely yours,

Estela Galván
