# Supplementary material for: Cell death and biomass reduction in biofilms of multidrug resistant extended spectrum β-lactamase-producing uropathogenic Escherichia coli isolates by 1,8-cineole
Source: PLoS One. 2020 Nov 5;15(11):e0241978. doi: 10.1371/journal.pone.0241978 (PMC7644070; doi:10.1371/journal.pone.0241978)
Supplement: Attachment — Submitted filename: Galvan R2 - Response to Reviewers.docx [file pone.0241978.s002.docx]

Estela M. Galvan, Ph D
Lab. of Bacterial Pathogenesis

Centro de Estudios Biomedicos, Biotecnologicos,

Ambientales y Diagnostico (CEBBAD)

Universidad Maimonides
Hidalgo 775

C1405BWE-Buenos Aires, Argentina

July 20, 2020

Dear Monica Cartelle Gestal, PhD

Academic Editor

PLOS ONE

Please find enclosed our revised version of the manuscript number PONE-D-20-04063R1 entitled “Cell death and biomass reduction in biofilms of multidrug resistant extended spectrum β-lactamase-producing uropathogenic *Escherichia coli* isolates by 1,8-cineole” for your consideration. It has been revised and changes have been made for each specific comment of the reviewers, as addressed below (modifications are indicated, referring to line numbers in the Marked Up Manuscript file).

**Reviewer #2**: Second review of the proposed plos one article

***General comment:*** The authors have made several key improvements to the English as evident by the tracked changes version, however there are a number of flaws still remaining that will need addressing. Plos One does not copy edit so they must be fixed before acceptance.

***Author response to general comment:*** We addressed all marked flaws. We consider that now the whole manuscript has proper English usage.

***Major concern #1:*** How much of the reduction is due to the presence of tween 80? Previous work using the same compound did not make use of the surfactant. Why was it used and why was it not controlled for?

On line 219 the authors make mention of reduced bacterial growth. They should present this data. Did they do a tween negative control? If not, they should.

Line 389-392: has to be expanded significantly, currently other published work shows a much higher level for both MIC and bactericidal of the oil. What rational do the authors have for this discrepancy?

***Author response #1:*** Tween 80, as well as DMSO, are commonly used to increase solubility of lipophilic molecules, such as essential oils and their individual compounds, in aqueous solutions (Man and Markham, 1998; Ojeda-Sana *et al*., 2013; Kwiatkowski *et al*., 2019). The effect of Tween 80 (0.5 %) was tested, and it did not show any detrimental effect neither on planktonic growth (MIC determinations) nor on biofilms (biomass, cell viability, cell detachment). We have now rewritten Material and methods section to clarify this issue (see L 143-154 and L164-170).

Regarding the reduction of bacterial growth mentioned on line 219, we now presented the data in Table 2 (see L253). As explained above, tween negative controls have been performed.

As requested, additional published work regarding MIC and bactericidal effect of 1,8-cineole has been included and discussed in the context of our findings (see L457-479).

***Major concern #2:*** Given that the majority of clinical isolates did not make biofilms the authors must address the rational for using a biofilm disrupting treatment. I see three possibilities, and all should be discussed. Either the medium (M9) is not sufficient to recapitulate the clinical environment, reducing the enthusiasm for this work. Or the presence of biofilm forming strains in actual clinical samples is limited raising question about the usefulness of this treatment. Third, perhaps samples collected from clinical are bias for not biofilm forming as they would presumably be easier to collect. I would ask the author to at least address point 1 and 2 and remove all work related to the strain that did not produce biofilms.

***Author response #2:*** We addressed this reviewer’s concern by adding a paragraph in the Discussion section (see L395-407). Additionally, strains that did not produce biofilm were removed from Fig 1.

***Major concern #3***- The authors use 3 different methods of approximating biofilms. Crystal violet, CFUs, and confocal live dead staining. All 3 methods gave wildly different levels of reduction. For example, why did the CFU have 3-4 log reduction while the confocal had around a 1-2 log fold? Why don’t these methods agree? This I find further concerning as the MIC does not agree with the biofilm data. I would revise the section from line 364-379 to address this problem.

***Author response #3:*** For Ec AM7 biofilms, our results showed 2-4 log cell viability reduction by CFU counts and ⁓ 2 log reduction by confocal live/dead microscopy. These results are within the inter-experimental variation observed. In addition, while CFU counting assesses viable cells in the whole biofilm sample, confocal microscopy explores a limited number of microscopic fields of view.

Regarding the reviewer´s concern that MIC does not agree with the biofilm data, we consider that this is an important finding of the present work. Notably, the phytochemical showed better antimicrobial effect on biofilms compared to planktonic cells. This is in agreement with a recent report (Schurmman et al, 2019). We now better discuss this issue in the manuscript (see L465-475).

***Major concern #4***- Section starting on line 309. Are the cells actually released from the biofilm or are they just growing planktonically? If they are released than according to fig 5A the ethanol treatment is better at releasing cells. If they are just growing planktonically this section needs to be revised.

***Author response #4:*** To address this reviewer´s comment we now included additional experimental data in Fig 5A. We also added a paragraph in the Results section regarding this issue (see L360-364 and L380).

***Minor concerns***

***#1.*** Line 35: English. **Response:** Modified (see L35).

***#2.*** Line 38: English. **Response**: Modified (see L39).

***#3.*** Line 63: Needs a citation. **Response:** As requested, a citation has now been included (see L63).

***#4.*** Line 68: needs a citation. **Response:** As requested, a citation has now been included (see L68).

***#5.*** Line 74: why differentiate between fungi and yeast? **Response:** We regret this mistake and now the sentence was re-written (see L74).

***#6.*** Line 83-84 I would remove the QS section. It adds nothing. **Response:** As requested, this sentence has been removed (see L83-84).

***#7.*** Line 116: English in two instances. Freshly streaked doesn’t mean anything and you do not streak *in* agar but *on* it. **Response:** This sentence has been now modified (see L116).

***#8.*** Line 117: what is the volume used for the overnights? **Response:** The requested information has now been added (see L117).

***#9.*** Line 136: Why did you only use duplicates here? This data is not robust for statistically analysis. Please state all replicate numbers in figure legends. **Response:** Experiments were done in biological triplicate and technical duplicates were done. We now clarified this information in Materials and Methods section (see L129-131, L138-139). This data is robust for statistical analysis, as detailed in the raw data. All biological replicate numbers are stated in figure legends.

***#10.*** Line 146: space between tween 80 is missing. **Response:** Corrected (see L162).

***#11.*** Line 149: state what the vehicle is and how much was used. **Response:** Modified as requested (see L164-170).

***#12.*** Line 152: was only 1 assay done as the sentence implies? Or should it be biofilm biomass and cell viability? **Response:** This sentence has been corrected (see L171).

***#13.*** Line 155: English. **Response:** Modified (see L175).

***#14.*** Line 182: English. **Response:** Modified (see L202-203).

***#15.*** Line 216: table say equal or greater to, but text suggests it is sensitive to 2%. **Response**: For clarity, the corresponding sentence was re-written (see L241-245).

***#16.*** Line 236: English. **Response:** Modified (see L270-274).

***#17.*** Line 248: and other parts, the authors say at least 3 replicates, are there uneven tests? What did the authors do to reduce the P-hacking of sampling at different rates per treatment? Or is this a case of an English mistake? **Response:** We apologize for any English mistake. We now re-write these sentences to better explain what has been done (see L283, L288-289, L307-308, L327, L383).

***#18.*** Line 254-255: Why does biomass (Fig 1) not correlate with figure 3? **Response:** Fig 1 shows biofilm biomass assessed by crystal violet whereas fig 3 shows cell viability of biofilms assessed by colony forming units (CFU). Whereas the crystal violet dye detects negatively-charged molecules in the biofilm (such as polysaccharides in the biofilm matrix, and lipopolisaccharides from bacterial cells), the CFU counting is the standard technique to detect viable cells. Because each technique detects different characteristics of the biofilm, no strict correlation between them is expected. Anyhow, for example, biofilm biomass and CFU numbers were higher for Ec ATCC25922 biofilms than for Ec AM10 biofilms.

***#19.*** Line 260: English. **Response:** Modified (see L301-302).

***#20.*** Line 282: What is the replicate number for data in table 3. **Response**: The requested information has now been added (see L327).

***#21.*** Line 288 English. **Response:** Modified (see L332-333).

***#22.*** Line 316 & 318: give exact numbers. **Response:** Modified as requested (see L362).

***#23.*** Line 366: English. **Response:** Modified (see L430).

***#24.*** Line 411: English. **Response:** Modified (see L502).

**Reviewer #3**

***Specific comment #1:*** The authors evaluate biofilm formation of ten E. coli isolates. Only one of them presented a substantial biofilm formation and two a mild formation. Raw data presented supports these conclusions. However, data dispersion of biofilm formation in biofilm-forming strains is unusual. Authors should discuss these anomalies.

***Author response #1:*** As requested, we now discuss this issue in the manuscript (see L227-230).

***Specific comment #2:*** Authors should analyze the low frequency of biofilm-forming isolates in the context of

evaluating a possible biofilm inhibition treatment.

***Author response #2:*** As requested, we now discuss this findings (see L396-408).

***Specific comment #3:*** Fig2A. The authors show cell viability on biofilms when they increased 1,8 cineole

concentration. In raw data authors present five assays for treatment experiment but only one for control. Although differences between treatment and control with concentrations > 0.5% are important, authors must demonstrate this with a test. I don´t think the curve representation is the best suitable for these result.

Authors can consider present results in two parts: ethanol in extracts is not detrimental for survival and on the other side, dependence of viability to 1,8 cineole concentration compared to non 1,8 cineole (first column on raw data).

***Author response #3:*** As requested, the concentration-dependent data in the old Fig. 2A is now displayed in two parts: ethanol effect in one panel (A) and 1,8-cineole effect in other panel (B). We additionally performed an ANOVA test to analyze significance of differences in cell viability after treatment (see new Fig2).

***Specific comment #4:*** Table 3. The dispersion values between assays are noteworthy. Although differences are

significant authors should address why they have such differences between assays. Dispersion was not observed in raw data for fig 1. I assume that some disruption of biofilm was produced during washes during treatment.

***Author response #4:*** We have now included a sentence to discuss variations between assays (see L316-318).

***Specific comment #5:*** Authors described viability of detached cells arguing “It has been postulated that a good

antibiofilm agent should not only attack bacteria into the biofilm but also display an action against biofilm-released cells”. The results is in concordance with high rate of dead cells on biofilm. It would be more interesting and will significantly improve the impact of the work to investigate if a post-treatment biofilm is able to growth.

***Author response #5:*** We agree with the reviewer´s comment regarding post-treatment biofilms. Even though this experiment is beyond the scope of the present work, we now mention in the manuscript their importance (see L435-436).

***Specific comment #6:*** Authors did not demonstrate that high biofilm biomasses yielded by strains are consequence of thicker extracellular matrix. Statement in L388-L393 should be modified.

***Author response #6:*** The requested statement has been modified (see L415-416).

We thank the reviewers and editors for their constructive comments and suggestions. We believe that the revised manuscript is now acceptable for publication in PlosOne.

Sincerely yours,

Estela Galván
