# Supplementary material for: Cell death and biomass reduction in biofilms of multidrug resistant extended spectrum β-lactamase-producing uropathogenic Escherichia coli isolates by 1,8-cineole
Source: PLoS One. 2020 Nov 5;15(11):e0241978. doi: 10.1371/journal.pone.0241978 (PMC7644070; doi:10.1371/journal.pone.0241978)
Supplement: Attachment — Submitted filename: GalvanR3 -Response to Reviewers.docx [file pone.0241978.s003.docx]

Estela M. Galvan, Ph D
Lab. of Bacterial Pathogenesis

Centro de Estudios Biomedicos, Biotecnologicos,

Ambientales y Diagnostico (CEBBAD)

Universidad Maimonides
Hidalgo 775

C1405BWE-Buenos Aires, Argentina

September 24, 2020

Dear Monica Cartelle Gestal, PhD

Academic Editor

PLOS ONE

Please find enclosed our revised version of the manuscript number PONE-D-20-04063R2 entitled “Cell death and biomass reduction in biofilms of multidrug resistant extended spectrum β-lactamase-producing uropathogenic *Escherichia coli* isolates by 1,8-cineole” for your consideration. It has been revised and changes have been made for each specific comment of the reviewers, as addressed below (modifications are indicated, referring to line numbers in the Marked Up Manuscript file).

**Reviewer #3**:

***General comment:*** Although most of the comments have been addressed, there are still a number of flaws that need to be addressed.

***Concern #1:*** The authors cite an article by Kragh et al to justify the stochastic variation in the biofilm results for the 72-hour biofilm and discourage the use of the CV staining method to draw conclusions. However, variations in biomass quantification were observed by microscopy (CV results were not shown) and were not as large as presented in this manuscript. Authors must discuss the comment more extensively than citing the article by Kragh et al.

***Author response #1:*** As requested, we have now discuss more extensively the experimental variables that could account for the large biomass variations observed among biological replicates in the stronger biofilm-producer strains, particularly in the reference strain Ec ATCC25922 (see L231-242).

***Concern #2:*** Specific comment # 5. I disagree with the authors. I think the suggested experiment would improve significantly impact of work. The authors present a new drug capable of altering and killing the cells presented in the biofilm life form. It is important to determine if the cells can grow.

***Author response #2:*** As requested, new experimental data related to whether a post-treatment biofilm is able to regrow have been added (see L41-42, L169-176, L391-409 –including new Table 4-, L453-458).

We thank the reviewers and editors for their constructive comments and suggestions. We believe that the revised manuscript is now acceptable for publication in PlosOne.

Sincerely yours,

Estela Galván
